# Supplementary material for: The interactome of intact mitochondria by cross-linking mass spectrometry provides evidence for coexisting respiratory supercomplexes
Source: Mol Cell Proteomics. 2017 Dec 8;17(2):216–32. doi: 10.1074/mcp.RA117.000470 (PMC5795388; doi:10.1074/mcp.RA117.000470)
Supplement: Supplemental Data [file supp_RA117.000470_133922_0_supp_23978_fzffwf.zip › spectra_annotation/mito_DR_spectra_annotation/306-1-1-1-5-1.pdf]

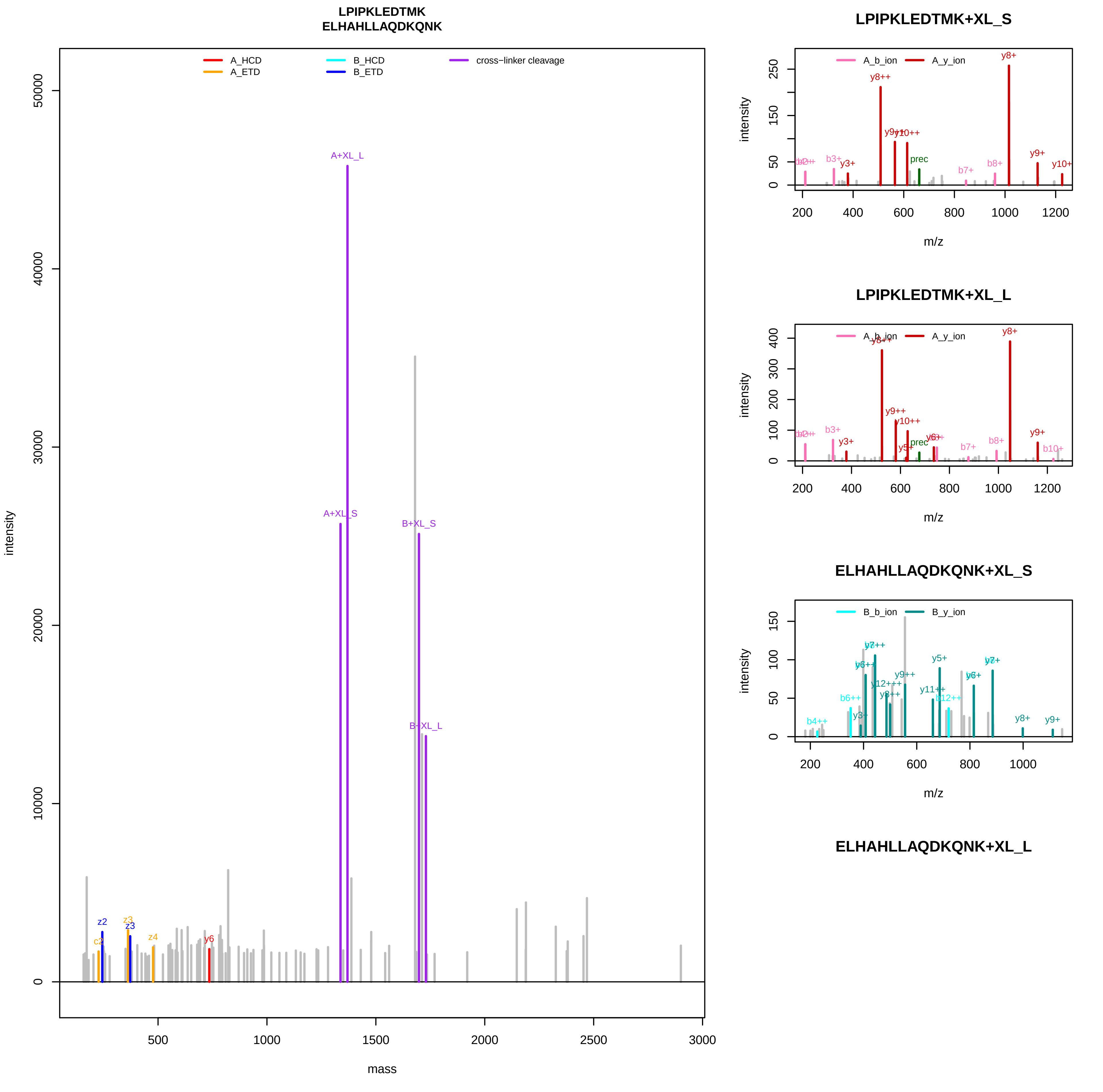

LPIPKLEDTMK
ELHAHLLAQDKQNK
A_HCD
A_ETD
B_HCD
B_ETD
cross−linker cleavage
A+XL_L
A+XL_S
B+XL_S
B+XL_L
z2
c2
z3
z3
z4
y6
intensity
mass
0
10000
20000
30000
40000
50000
500
1000
1500
2000
2500
3000
LPIPKLEDTMK+XL_S
A_b_ion
A_y_ion
y8+
y8++
y9++
y10++
prec
b3+
y3+
b7+
b8+
y9+
y10+
intensity
m/z
LPIPKLEDTMK+XL_L
A_b_ion
A_y_ion
y8+
y8++
y9++
y10++
b3+
y3+
prec
b7+
b8+
y9+
b10+
intensity
m/z
ELHAHLLAQDKQNK+XL_S
B_b_ion
B_y_ion
y7++
y6++
y5+
y7+
y6+
y9++
y12+++
y8++
y11++
b12++
b6++
b4++
y3+
y8+
y9+
intensity
m/z
ELHAHLLAQDKQNK+XL_L
